# Neutrophil-to-albumin Ratio as a Prognostic Factor in Patients with Acute Ischemic Stroke

**DOI:** 10.2174/0115672026328594240614080241

**Published:** 2024-06-24

**Authors:** Jiajia Bao, Yang Zhang, Mengmeng Ma, Jian Wang, Xin Jiang, Jian Guo, Li He

**Affiliations:** 1 Department of Neurology, West China Hospital, Sichuan University, Chengdu, China

**Keywords:** Neutrophil-to-albumin ratio, NAR, inflammatory biomarker, AIS, prognosis, receiver operating characteristic, serum biomarker

## Abstract

**Background:**

Neutrophil-To-Albumin Ratio (NAR) is a novel inflammatory biomarker. However, the potential prognostic value of NAR in acute ischemic stroke (AIS) remains unclear. This study aimed to evaluate whether NAR levels correlated with the 3-month modified Rankin scale (mRS) in patients with AIS.

**Methods:**

AIS patients were included in this retrospective study. NAR was calculated as the ratio of absolute neutrophil count to serum albumin level. Logistic regression analyses were used to investigate the effect of NAR on 3-month mRS of AIS. The predictive values of NAR, albumin level, and neutrophil count were compared utilizing receiver operating characteristic (ROC) curves. Moreover, subgroup analyses and interaction tests were conducted to evaluate the consistency of NAR’s effect on AIS prognosis.

**Results:**

Of the 780 patients included, 403 (51.67%) had a poor clinical outcome (mRS 3-6) at 3 months. NAR was independently correlated to 3-month poor functional outcome after adjusting for confounders (Odds ratios (OR), 9.34; 95% confidence intervals (CI), 1.09 to 80.13; *p* =0.0417). Subgroup analysis showed a relative effect consistent with the overall population results, and no statistical interactions were found in the subgroups (all *p* for interaction > 0.05). The ROC curve showed that the prognosis-related cutoff value for NAR was 0.123, with corresponding specificity and sensitivity of 53.55% and 63.94%, respectively. When comparing the predictive power, NAR (0.590; 95%CI 0.549–0.630) exhibited the highest area under the curve (AUC) of ROC compared to neutrophils (0.584; 95%CI 0.543–0.624) and albumin (0.540; 95%CI 0.500–0.581).

**Conclusion:**

There is a positive relationship between NAR levels and 3-month poor functional outcomes in AIS patients, supporting the potential of NAR as a readily available and economic serum biomarker for the early identification of AIS prognosis. Further studies are required to validate the prognostic value and clinical utility of the NAR.

## 1 INTRODUCTION

Stroke remains a leading cause of death and disability in the world [1, 2] Acute ischemic stroke (AIS) accounts for approximately 70% of all incident strokes, which contributes to a significant global disease burden [1-3]. Considering the high disability rate of AIS, continuous research to find convenient and effective prognostic biomarkers is crucial for early detection of individuals at risk for poor clinical outcomes. It could help inform clinical decisions in AIS patients [4].

Inflammation plays a pivotal role in AIS, which is triggered by ischemic brain injury and involves the activation of peripheral immune cells. As a result, it has effects on worse brain repair and AIS outcomes [5-9]. Increasing evidences suggested that some serum inflammatory biomarkers, measurable in peripheral blood, have prognostic value in AIS, such as the lymphocyte to monocyte ratio (LMR) and neutrophil to lymphocyte ratio (NLR) [6, 7, 10-14]. The neutrophil-to-albumin ratio (NAR), a novel inflammatory biomarker combining absolute neutrophils count and albumin, has been used to assess the prognosis of various diseases, including aneurysmal subarachnoid hemorrhage [15-17], hemorrhagic stroke [18], lung cancer [19, 20], rectal cancer [21], colorectal cancer [22], and non-alcoholic fatty liver disease [23]. Notably, NAR has demonstrated a better prognostic value compared to neutrophil or serum albumin levels in these diseases. For this reason, NAR may also be a potential biomarker for AIS prognosis. Although a previous study suggested a correlation between NAR and mortality in hemorrhagic stroke patients [18], the association between NAR and the prognosis of patients with AIS remains unknown.

As a result, we designed a retrospective cohort study with the purpose of investigating the association between NAR levels and the prognosis of AIS. Moreover, subgroup analyses and interaction tests were performed to assess the consistency of NAR’s effect on AIS prognosis. Our findings may be useful in helping clinicians identify AIS patients at a high risk of poor functional outcomes in clinical practice.

## 2 METHODS

### 2.1 Ethical Statement

The study was performed in compliance with the STROBE (Strengthening the Reporting of Observational Studies in Epidemiology) declaration [24]. The Ethical Review Committee of the West China Hospital of Sichuan University approved this research (2019(319)). Because this is a retrospective study collecting de-identified data, the requirement of obtaining informed consent was waived.

### 2.2 Study Design and Patient Population

**Fig. (1):**
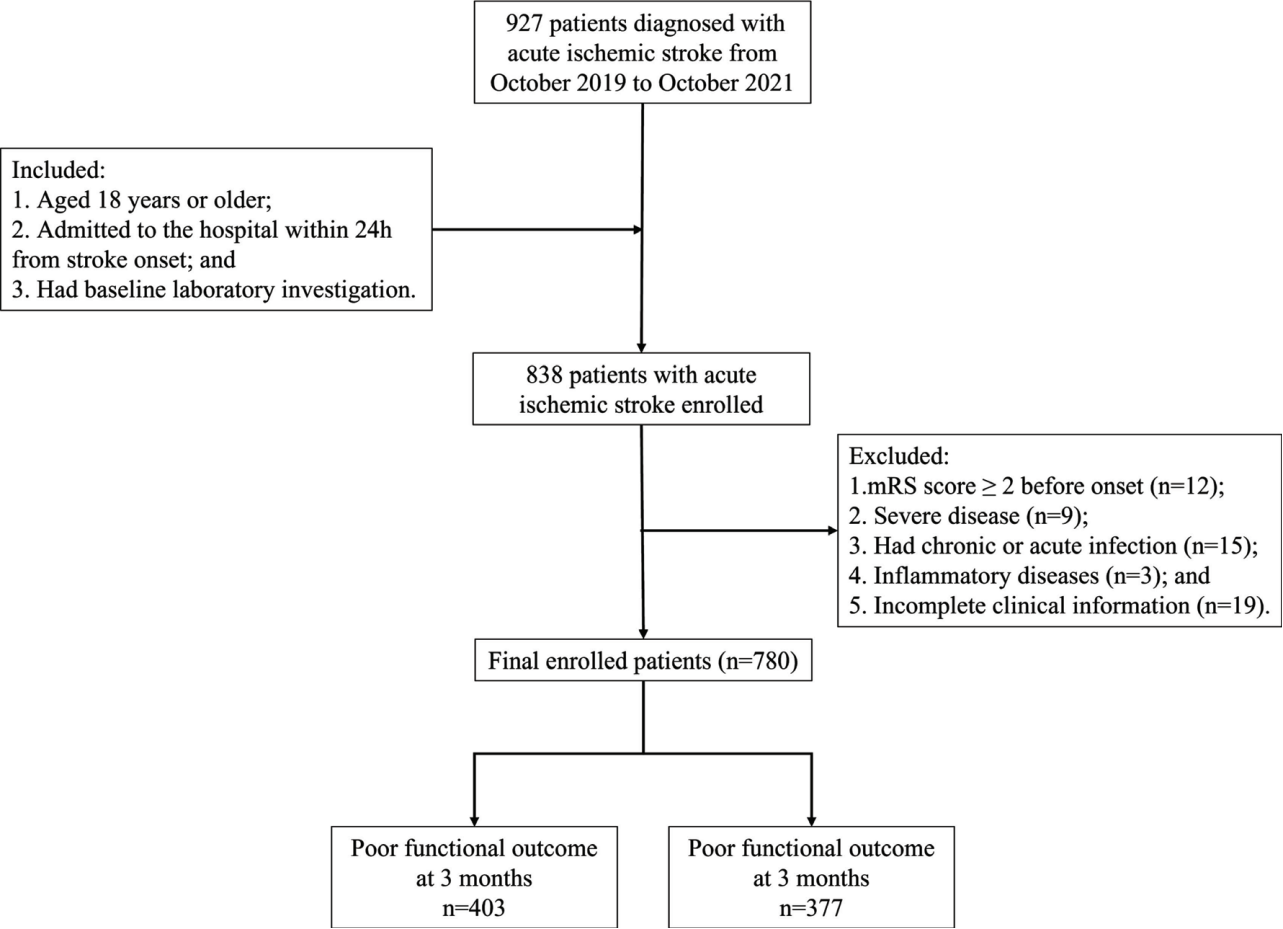
Flowchart of the patient selection. A total of 780 patients were included in this study. **Abbreviation:** mRS=modified Rankin scale.

We retrospectively screened and selected consecutive patients with AIS between October 2019 and October 2021 from a prospectively maintained database at the Neurology Department, West China Hospital, Sichuan University. AIS diagnosis followed the World Health Organization stroke diagnosis criteria, which needed neuroimaging evidences. Patients who fulfilled all of the following criteria were included in this study: (1) aged 18 years or older, (2) admitted to the hospital within 24 hours of the stroke onset, and (3) underwent baseline laboratory examination. The following were the exclusion criteria: (1) modified Rankin scale (mRS) score ≥ 2 before onset; (2) infection within 2 weeks of AIS onset; (3) patients with conditions like chronic infection, hematological diseases, recent major surgery or trauma, coagulopathy, history of substance abuse, or other severe diseases; (4) incomplete information or missing follow-up. Overall, 780 patients with AIS who fulfilled the inclusion criteria were enrolled in this study (Fig. **1**).

### 2.3 Data Collection and Definitions

Demographic information, vascular risk factors, pre- and post-AIS therapy, baseline National Institutes of Health Stroke Scale (NIHSS) score, laboratory data, and angiographic data were taken from the hospital's electronic medical records. Hemorrhagic transformation (HT) was defined as priorly reported [25]. A blinded neuroradiologist reviewed the CT images to confirm the occurrence of HT. The Trial of ORG 10172 in Acute Stroke Treatment (TOAST) classification was used to evaluate the stroke subtype [26]. Stroke severity was categorized by the NIHSS score on admission [27]. Body mass index (BMI) was classified in the way that was previously reported [28]. Moreover, patients aged 65–79 years were referred to ‘elderly’, while those aged is over 80 years referred to as ‘very old’, as previously described [29]. Venous blood samples were acquired within 24 hours of admission in accordance with standard institutional guidelines. NAR was calculated as the ratio of absolute neutrophil count to serum albumin level [15-17].

### 2.4 Clinical Outcome

The mRS was used to define clinical outcomes at 90 days *via* phone or face-to-face interviews during clinical follow-up. The definition of poor clinical outcome was defined as an mRS score ranging from 3 to 6, whereas a good clinical outcome was defined as an mRS score within the range of 0 to 2.

### 2.5 Statistical Analysis

The patient characteristics were presented using descriptive statistics. Categorical and ordinal data are expressed as frequencies or percentages, while continuous data are expressed as mean ± standard deviation (SD) or median (interquartile range [IQR]). The t-test or Mann–Whitney U-test was utilized to analyze the continuous data, and the chi-square test or Fisher’s test was utilized to analyze the categorical data. Correlation between the poor clinical outcome and prognostic factors was initially analyzed using univariate logistic regression analysis. Subsequently, we developed three models in multivariate logistic regression analysis to determine the independent associations between NAR and poor clinical outcome: the non-adjusted model, no covariates were adjusted; model 1, where only demographic data were adjusted; model 2, where covariates in model 1 and other potential confounders (with a *p*-value less than 0.05 at univariate analysis and clinically relevant factors) were adjusted. To compare the predictive values of neutrophil counts, albumin, and NAR, we calculated the area under the curve (AUC) of the receiver operating characteristic (ROC) curve. Additionally, we analyzed the best cut-off for NAR using the ROC curve. To evaluate the robustness of the relationship between NAR and 3-month functional outcomes in different subgroups, stratified analysis and interaction tests were performed. The likelihood ratio test was conducted after testing for effect modification of subgroup indicators. The Results were presented as odds ratios (OR), 95% confidence intervals (CI) and *p*-value.

All analyses were performed using the statistical package R (4.2.0 version) and IBM SPSS Statistics software (version 22). All statistical tests were two-sided, and statistical significance was defined as *p* value less than 0.05.

## 3 RESULTS

### 3.1 Baseline Characteristics

Finally, 780 patients were included in this study (Fig. **1**). The demographic data of all patients is shown in Table **1**. Of the 780 patients, 403 (51.67%) had poor clinical outcomes (mRS 3-6) at 3 months. The mean age of all patients was 69.44 ± 13.14 years old, comprising 424 (53.88%) male and 363 (46.12%) female. The mean age was 65.79 ± 13.45 years and 73.02 ± 11.76 years with good clinical outcome or poor clinical outcome at 3 months, respectively. The median onset-to-admission time was 180 (113-282) mins, and the median baseline NIHSS score was 11 (5-17). Patients with poor clinical outcomes were older, had higher baseline NIHSS scores, longer onset-to-admission times, higher levels of NAR, a higher proportion of females, and had a history of prior hypertension, diabetes mellitus (DM), atrial fibrillation (AF), coronary heart disease (CAD), current smoking, anterior occlusion, endovascular treatment (EVT), and HT. In contrast, they had a lower proportion of prior statin use, posterior circulation occlusion, thrombolysis, antiplatelet treatment after admission, anticoagulant treatment after admission, and statin treatment after admission compared to patients with good clinical outcomes at 3 months.

### 3.2 Logistic Regression Analysis of AIS Prognosis Risk Factors

Table **2** shows the results of the univariate and multivariate logistic regression analysis of variables in the groups with good and poor clinical outcomes. Univariate analysis shown that age (OR, 1.05; 95%CI, 1.03 to 1.06; *p* <0.0001), gender (OR, 0.42; 95%CI, 0.32 to 0.56; *p* <0.0001), DM (OR, 1.62; 95%CI, 1.09 to 2.41; *p* =0.0178), hypertension (OR, 1.48; 95%CI, 1.12 to 1.97; *p* =0.0063), AF (OR, 2.01; 95%CI, 1.44 to 2.79; *p* <0.0001), CAD (OR, 1.73; 95%CI, 1.13 to 2.64; *p* =0.0112), current smoking (OR, 0.57; 95%CI, 0.42 to 0.76; *p*=0.0002), baseline NIHSS score (OR, 1.18; 95%CI, 1.15 to 1.21; *p* <0.0001), prior statins use (OR,0.54; 95%CI, 0.32 to 0.92; *p* =0.0248), occlusive site (OR, 0.36; 95%CI, 0.24 to 0.56; *p* <0.0001), CE (OR, 1.75; 95%CI, 1.27 to 2.43; *p* =0.0007), SAO (OR, 0.28; 95%CI, 0.12 to 0.62; *p* =0.0020), baseline neutrophil (OR, 1.06; 95%CI, 1.01 to 1.11; *p* =0.0106), baseline lymphocyte (OR, 0.82; 95%CI, 0.70 to 0.97; *p* =0.0229), NAR (OR, 12.11; 95%CI, 1.89 to 77.67; *p* =0.0086), PLT (OR, 1.00; 95%CI, 0.99 to 1.00; *p* =0.0114), thrombolysis (OR, 0.69; 95%CI, 0.51 to 0.93; *p* =0.0164), EVT (OR, 1.45; 95%CI, 1.06 to 1.99; *p* =0.0193), antiplatelets treatment after admission (OR, 0.34; 95%CI, 0.24 to 0.48; *p* <0.0001), anticoagulants treatment after admission (OR, 0.62; 95%CI, 0.43 to 0.88; *p* =0.0073), statins treatment after admission (OR, 0.33; 95%CI, 0.21 to 0.52; *p* <0.0001), HT (OR, 6.21; 95%CI, 3.57 to 10.81; *p* <0.0001) were correlated with 3-month poor clinical outcome of AIS patients, while other variables had no significant relationship with AIS prognosis. After adjusting for potential confounders (model 1 adjusted for age, gender, hypertension, DM, CAD, smoking, and baseline NIHSS score; model 2 adjusted for model 1 plus stroke subtypes, HT, prior statins treatment, anticoagulants use after admission, statins use after admission, antiplatelets use after admission, occlusive site, and reperfusion therapy), the age (OR, 1.03; 95%CI, 1.02 to 1.05; *p* <0.0001), male (OR, 0.56; 95%CI, 0.35 to 0.88; *p* =0.0127), baseline NIHSS score (OR, 1.18; 95%CI, 1.14 to 1.22; *p* <0.0001), prior statins use (OR, 0.38; 95%CI, 0.19 to 0.79; *p* =0.0096), NAR (OR, 9.34; 95%CI, 1.09 to 80.13; *p* =0.0417), thrombolysis (OR, 0.46, 95%CI (0.31, 0.70), *p* =0.0002), endovascular treatment (OR, 0.52; 95%CI, 0.33 to 0.82; *p* =0.0046), anticoagulants use after admission (OR, 0.43; 95%CI, 0.26 to 0.72; *p* =0.0011), antiplatelets use after admission (OR, 0.51; 95%CI, 0.31 to 0.83; *p* =0.0069), and HT (OR, 3.62; 95%CI, 1.86 to 7.05; *p* =0.0002) were statistically significant potential factors of AIS prognosis (Table **2**).

### 3.3 Independent Prognostic Effect of NAR and Stratified Analysis

To assess whether the correlation between 3-month poor functional outcome and NAR in AIS patients was consistent in different subgroups, we further conducted stratification analysis and interaction tests. As shown in Fig. (**2**), the subgroup analysis showed a relative effect consistent with the overall population results, and the clinical features were well-balanced. No significant interactions were found in the subgroups, including age (≤65 *vs*. 65–79 *vs*. ≥80 years), sex (female *vs*. male), stroke severity (mild-to-moderate *vs*. severe), stroke subtypes (CE *vs*. non-CE), HT (no *vs*. yes), current smoking (no *vs*. yes), occlusive site (anterior circulation infarction *vs*. posterior circulation infarction), thrombolysis (no *vs*. yes), EVT (no *vs*. yes), anticoagulant treatment after admission (no *vs*. yes), and antiplatelet treatment after admission (no *vs*. yes) (all *p* for interaction > 0.05).

### 3.4 ROC Curve Analysis

As shown in Fig. (**3**), when comparing the predictive power of neutrophils and albumin, NAR (0.590; 95%CI, 0.549 to 0.630) displayed the highest AUC value compared with neutrophils (0.584; 95%CI, 0.543 to 0.624) and albumin (0.540; 95%CI, 0.500 to 0.581). The best cut-off threshold of NAR for distinguishing between good and poor clinical outcomes at 3 months of AIS was 0.123, with corresponding specificity and sensitivity of 53.55% and 63.94%, respectively.

## 4 DISCUSSION

In the present study, we found that the NAR level had a positive correlation with poor 3-month functional outcomes in AIS patients, which was more effective in predicting the outcome of AIS than neutrophil or serum albumin levels alone. These results suggest that NAR could serve as a potential biomarker for AIS prognosis. To the best of our knowledge, this is the first study to focus on the relationship between NAR and prognosis in AIS patients.

AIS is a severe cerebrovascular disease with high morbidity and mortality [1]. It is evidenced that neuroinflammatory response is implicated in the pathogenesis and progression of AIS [30]. NAR, combining neutrophil counts and serum albumin count, is a novel and convenient available biomarker for systemic inflammation status and has been proven to be linked to major adverse events, such as aneurysmal subarachnoid hemorrhage and lung cancer [15-23]. Whether the NAR levels related to the increased risk of unfavorable clinical outcomes has not been previously described in AIS patients. Our results are the first to reveal that NAR is associated with AIS prognosis. Specifically, we observed that the risk of poor 3-month outcomes in patients with AIS increased by more than 9-fold for every one-unit increase in NAR level. There were no significant interactions between NAR and potential confounders in subgroup analysis, suggesting a stable correlation between NAR and AIS prognosis.

This elevated NAR may result from an increase in neutrophils, a decrease in serum albumin, or a combination of both. Indeed, several studies have reported that neutrophils are considered the first responders to stroke [31, 32]. Damage to neutrophil homeostasis has a deleterious effect on AIS by influencing systemic inflammation and the blood-brain barrier (BBB) [30]. For example, the recruitment of neutrophils in the ischemic brain area could lead to microvascular obstruction and brain blood flow of microvessel reduction, thereby contributing to the aggravation of brain injury [33, 34]. Recent studies have indicated that activated neutrophils release neutrophil extracellular traps (NETs) composed of double-stranded DNA, histones, and granule proteins. These DNA extracellular networks are decorated with histones and granular proteins [35]. Accumulating evidence has revealed that the involvement of NETs leads to impairment of neurological function due to BBB breakdown and thrombosis after ischemic damage [36]. Taken together, neutrophil infiltration could exacerbate brain injury and have a virtual role in inflammatory responses during AIS progression, as consistent with our finding that neutrophils correlate with a 3-month poor functional outcome post-AIS.

Although serum albumin levels were not independently related to AIS prognosis in the current research, elevated NAR in AIS patients with poor clinical outcomes may indicate a potential difference in serum albumin levels between patients with poor and good clinical outcomes. It is possible that the neuroprotective effect of serum albumin accounts for this potential difference [37]. As Tomasz Dziedzic *et al*. found in their study, low serum albumin was demonstrated to be related to cortisol release and predisposes to hypercortisolemia in patients with AIS [33]. Likewise, prior results from animal experiments indicated that protein malnutrition increases pituitary-adrenocortical activity and results in excessive cortisol release [38, 39]. Notably, higher cortisol after AIS contributed to increased mortality and a higher risk of poor outcomes [40]. On the other hand, serum albumin could somewhat reflect nutrient status and protein-energy malnutrition after AIS was deleterious to the prognosis of patients with AIS [41]. Despite the fact that we did not observe the protective effect of albumin for AIS prognosis, a significant correlation between NAR and poor outcomes of AIS was detected in the present study. This finding also implies that NAR might have greater utility in predicting AIS outcomes than albumin levels alone.

More recently, numerous complete blood count-derived indices have been widely utilized to predict the prognosis and progression of AIS, owing to easier sample access and cost-effectiveness [13]. Nevertheless, it remains unclear which serum biomarkers predict AIS outcome with sufficient predictive value. Our results showed that NAR outperformed neutrophils and serum albumin alone in predicting the functional outcome of AIS, suggesting that NAR, as a combination index, is more likely to provide useful prognostic information. In line with this study, NAR was also superior to a single biomarker in predicting the prognosis of other diseases, such as aneurysmal subarachnoid hemorrhage [15-17]. Based on those observations, it is postulated that NAR may be a promising candidate for predicting the prognosis of AIS. This possibility merits further research to explore the potential clinical applications of NAR.

There were some limitations in the present study. First, since this was a retrospective single-center study, there is an inevitable selection bias to consider while interpreting our results. Despite statistically adjusting for potential confounders in multivariate and subgroup analyses to mitigate such effects, further research with larger and multicenter cohorts is warranted to validate and extend our findings. Second, the NAR was calculated using a single blood test. Given that blood cells are short-lived, serial testing might be better suited for obtaining more reliable information. Third, blood sample availability across a standardized time point (within 24 hours after admission), and the NAR and other serum biomarkers can fluctuate during this period. From this point of view, future studies involving prospective validation cohorts and serial monitoring of NAR are necessary to certify the predictive value of NAR and to investigate its dynamic changes at different stages of AIS. This will better assist clinicians in understanding the significance and utility of this biomarker in clinical practice.

## CONCLUSION

In conclusion, our study demonstrated that elevated NAR levels were related to a higher risk of poor functional outcomes in AIS patients. This finding supports the potential of NAR as a readily available and economical serum biomarker for early identification of AIS prognosis. Nonetheless, we were unable to determine whether NAR was the best serum biomarker for AIS prognosis. Thus, future studies are required to validate the prognostic value and clinical utility of NAR.

## AUTHORS’ CONTRIBUTIONS

JB contributed to the conception, design, statistical analysis, and drafting of the manuscript; JG and LH contributed to the design, supervision, and drafting of the study; and YZ, MM, JW, and XJ contributed to data collection. All authors reviewed and edited the manuscript and approved the final version.

## Figures and Tables

**Fig. (2):**
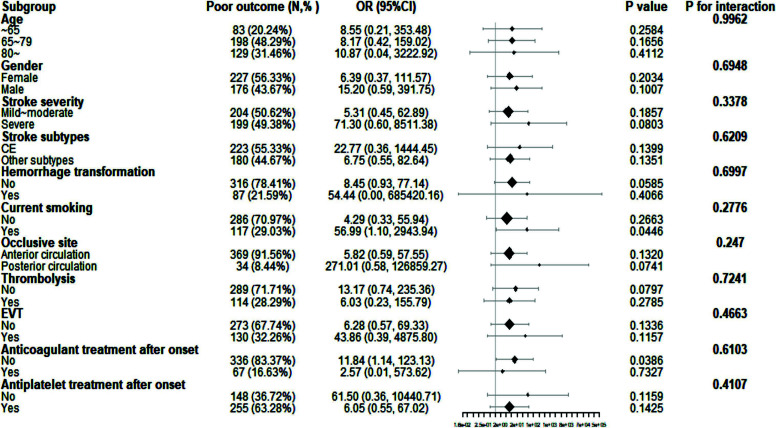
Subgroup analyses and interaction tests of the association between NAR and 3-month poor clinical outcome in AIS patients. No significant interactions were observed across subgroups, including age, gender, stroke severity, stroke subtypes, hemorrhagic transformation, smoking status, occlusion site, and treatments after admission. This suggested that the relationship between NAR and 3-month functional outcomes was stable across our study population. **Abbreviations:** OR=odds ratio; CI=confidence interval; EVT=endovascular treatment.

**Fig. (3):**
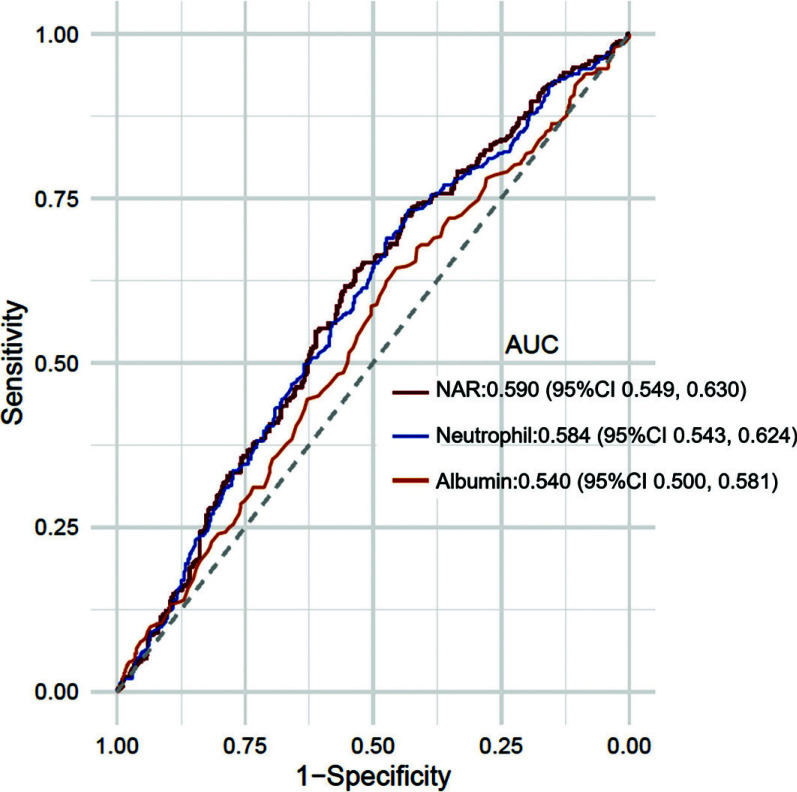
ROC curve and AUC showing the predictive ability of NAR, neutrophil, and albumin for 3-month poor clinical outcome in AIS patients. NAR demonstrated the highest AUC compared to either neutrophil or albumin levels alone, suggesting that NAR is more effective as a prognostic indicator. **Abbreviations:** NAR=Neutrophil-to-Albumin Ratio; ROC=receiver operating characteristic; AUC=area under the ROC curve.

**Table 1:** Demographics and clinical characteristics of the study population.

**-**	**Total** **(n=780)**	**Good Outcome** **(n=377)**	**Poor Outcome** **(n=403)**	***p* Value**
**Demographic Characteristics**				
Age, mean±SD, y	69.44 ± 13.14	65.79 ± 13.45	73.02 ± 11.76	<0.001
Sex, n(%)				<0.001
Female	363 (46.12%)	133 (35.28%)	227 (56.33%)	
Male	424 (53.88%)	244 (64.72%)	176 (43.67%)	
**Blood Pressure, Mean±SD, mmHg**				
SBP	147.25 ± 26.22	146.65 ± 25.24	148.24 ± 27.02	0.397
DBP	84.74 ± 16.92	84.64 ± 15.82	84.93 ± 17.86	0.812
BMI, mean±SD, kg/m2	23.17 ± 3.68	23.41 ± 3.56	22.91 ± 3.79	0.104
**Preexisting Conditions, n(%)**				
Hypertension	419 (53.24%)	182 (48.28%)	234 (58.06%)	0.006
Diabetes mellitus	121 (15.37%)	46 (12.20%)	74 (18.36%)	0.017
Atrial fibrillation	206 (26.18%)	73 (19.36%)	131 (32.51%)	<0.001
Coronary heart disease	106 (13.47%)	39 (10.34%)	67 (16.63%)	0.011
Dyslipidemia	17 (2.16%)	11 (2.92%)	6 (1.49%)	0.172
History of stroke	135 (17.15%)	64 (16.98%)	71 (17.62%)	0.813
Current smoking	277 (35.20%)	158 (41.91%)	117 (29.03%)	<0.001
**Medication Treatment before Onset**				
Antiplatelets, n(%)	77 (9.78%)	44 (11.67%)	32 (7.94%)	0.079
Anticoagulants, n(%)	74 (9.40%)	31 (8.22%)	43 (10.67%)	0.244
Statins, n(%)	61 (7.75%)	38 (10.08%)	23 (5.71%)	0.023
Antihypertensive, n(%)	279 (35.45%)	129 (34.22%)	150 (37.22%)	0.382
Hypoglycemic, n(%)	81 (10.29%)	32 (8.49%)	49 (12.16%)	0.093
**Clinical Variables**				
Baseline NIHSS, median (IQR)	11 (5-17)	5 (3-12)	15 (11-20)	<0.001
Occlusive site	-	-	-	<0.001
Anterior	676 (85.90%)	301 (79.84%)	369 (91.56%)	-
Posterior	111 (14.10%)	76 (20.16%)	34 (8.44%)	-
TOAST	-	-	-	<0.001
LAO, n(%)	255 (32.40%)	133 (35.28%)	120 (29.78%)	-
CE, n(%)	368 (46.76%)	141 (37.40%)	223 (55.33%)	-
SAO, n(%)	40 (5.08%)	32 (8.49%)	8 (1.99%)	-
OE, n(%)	17 (2.16%)	10 (2.65%)	7 (1.74%)	-
UE, n(%)	107 (13.60%)	61 (16.18%)	45 (11.17%)	-
Onset to admission time, median (IQR), min	180 (113-282)	166 (95-276)	185(120-287)	0.022
**Laboratory Parameters, Mean±SD**				
RBC, ×109/L	7.49 ± 34.18	7.79 ± 35.28	7.26 ± 33.47	0.832
WBC, ×109/L	14.46 ± 73.43	13.04 ± 71.51	15.88 ± 75.85	0.594
Neutrophil, ×109/L	6.39 ± 4.86	5.90 ± 3.55	6.68 ± 4.71	0.010
Albumin, g/L	42.32 ± 4.81	42.57 ± 4.65	42.08 ± 4.98	0.161
NAR	0.15 ± 0.12	0.14 ± 0.09	0.16 ± 0.13	0.009
Lymphocyte, ×109/L	1.62 ± 4.14	1.86 ± 5.78	1.37 ± 1.26	0.102
Platelet, ×109/L	168.13 ± 64.90	174.11 ± 63.51	162.23 ± 65.55	0.011
INR	1.20 ± 3.20	1.27 ± 4.40	1.14 ± 1.39	0.577
TC, mmol/L	6.16 ± 29.45	6.79 ± 33.86	5.58 ± 24.58	0.589
TG, mmol/L	1.83 ± 6.67	1.63 ± 1.21	2.03 ± 9.40	0.439
LDL, mmol/L	2.40 ± 1.11	2.43 ± 1.24	2.39 ± 0.97	0.613
HDL, mmol/L	1.59 ± 3.49	1.72 ± 4.93	1.47 ± 0.53	0.336
Serum glucose, mmol/L	8.05 ± 4.65	7.73 ± 4.06	8.33 ± 5.14	0.078
**Treatment after Admission, n(%)**				
Reperfusion therapy	-	-	-	-
Thrombolysis	254 (32.27%)	137 (36.34%)	114 (28.29%)	0.016
EVT	230 (29.22%)	93 (24.67%)	130 (32.26%)	0.019
Antiplatelets	575 (73.06%)	315 (83.55%)	255 (63.28%)	<0.001
Anticoagulants	160 (20.33%)	92 (24.40%)	67 (16.63%)	0.007
Statins	680 (86.40%)	349 (92.57%)	324 (80.40%)	<0.001
Antihypertensive	351 (44.60%)	161 (42.71%)	188 (46.65%)	0.268
Hypoglycemic	122 (15.50%)	51 (13.53%)	70 (17.37%)	0.139
**HT, n(%)**	103 (13.09%)	16 (4.24%)	87 (21.59%)	<0.001
**Death within 90 days, n(%)**	155 (19.87%)	-	155 (38.46%)	-

**Note:** *p value < 0.05; **Abbreviations:** SBP=systolic blood pressure; DBP=Diastolic blood pressure; BMI=Body Mass Index; NIHSS= National Institute of Health Stroke Scale; TOAST= Trial of ORG 10172 in Acute Stroke Treatment; LAO=large-vessel atherosclerosis; CE=Cardioembolic; SAO=Small-artery occlusion; OE=other etiology; UE=Undetermined etiology; NAR=neutrophil-to-albumin ratio; TC=total cholesterol; TG=triglyceride; LDL=low-density lipoprotein; HDL=high-density lipoprotein; EVT=endovascular treatment; HT=Hemorrhagic transformation.

**Table 2:** Univariate and multivariable logistic regression analysis for the risk factors associated with poor clinical outcome at 3 months.

**Characteristic**	**Univariate Regression**	**Multivariate Regression**	
		**Model 1#**	**Model 2†**
	**OR (95% CI), *p* Value**	**OR (95% CI), *p* Value**	**OR (95% CI), *p* Value**
**Demographic Characteristics**			
Age, y	1.05 (1.03, 1.06) <0.0001	1.03 (1.02, 1.05) <0.0001	1.03 (1.02, 1.05) <0.0001*
Male	0.42 (0.32, 0.56) <0.0001	0.57 (0.37, 0.88) 0.0116	0.56 (0.35, 0.88) 0.0127*
SBP	1.00 (1.00, 1.01) 0.3969	1.00 (0.99, 1.01) 0.9233	1.00 (0.99, 1.01) 0.8345
DBP	1.00 (0.99, 1.01) 0.8116	1.00 (0.99, 1.01) 0.3886	1.00 (0.99, 1.01) 0.9050
BMI, kg/m^2^	0.96 (0.92, 1.01) 0.1056	1.02 (0.96, 1.08) 0.4783	1.02 (0.96, 1.08) 0.5684
**Preexisting Conditions**			
Hypertension	1.48 (1.12, 1.97) 0.0063	1.28 (0.89, 1.83) 0.1833	1.23 (0.83, 1.81) 0.2987
Diabetes mellitus	1.62 (1.09, 2.41) 0.0178	1.46 (0.89, 2.39) 0.1322	1.36 (0.79, 2.35) 0.2660
Atrial fibrillation	2.01 (1.44, 2.79) <0.0001	1.10 (0.74, 1.66) 0.6330	1.34 (0.80, 2.23) 0.2637
Coronary heart disease	1.73 (1.13, 2.64) 0.0112	0.92 (0.54, 1.56) 0.7531	1.14 (0.64, 2.05) 0.6578
Dyslipidemia	0.50 (0.18, 1.37) 0.1800	0.79 (0.23, 2.74) 0.7106	0.95 (0.24, 3.72) 0.9410
History of stroke	1.05 (0.72, 1.52) 0.8129	0.88 (0.56, 1.40) 0.5878	1.05 (0.62, 1.77) 0.8543
Current smoking	0.57 (0.42, 0.76) 0.0002	1.13 (0.72, 1.78) 0.5953	1.25 (0.77, 2.03) 0.3641
**Medication Treatment before Onset**			
Antiplatelets	0.65 (0.40, 1.05) 0.0808	0.52 (0.28, 0.96) 0.0379	0.56 (0.23, 1.33) 0.1880
Anticoagulants	1.33 (0.82, 2.16) 0.2450	1.24 (0.65, 2.37) 0.5065	1.29 (0.61, 2.69) 0.5056
Statins	0.54 (0.32, 0.92) 0.0248	0.49 (0.25, 0.93) 0.0302	0.38 (0.19, 0.79) 0.0096 *
Antihypertensive	1.14 (0.85, 1.53) 0.3820	0.96 (0.61, 1.51) 0.8575	0.85 (0.52, 1.39) 0.5098
Hypoglycemic	1.49 (0.93, 2.39) 0.0947	1.58 (0.62, 3.99) 0.3379	1.89 (0.68, 5.25) 0.2210
**Clinical Variables**			
Baseline NIHSS	1.18 (1.15, 1.21) <0.0001	1.17 (1.14, 1.20) <0.0001	1.18 (1.14, 1.22) <0.0001*
Occlusive site	-	-	-
Anterior	-	-	-
Posterior	0.36 (0.24, 0.56) <0.0001	0.55 (0.32, 0.94) 0.0298	0.64 (0.37, 1.12) 0.1166
TOAST	-	-	-
LAO	-	-	-
CE	1.75 (1.27, 2.43) 0.0007	0.77 (0.49, 1.22) 0.2645	0.83 (0.49, 1.41) 0.4991
SAO	0.28 (0.12, 0.62) 0.0020	0.60 (0.24, 1.47) 0.2613	0.66 (0.26, 1.65) 0.3695
OE	0.78 (0.29, 2.10) 0.6178	1.13 (0.30, 4.21) 0.8605	0.99 (0.24, 4.07) 0.9908
UE	0.82 (0.52, 1.29) 0.3883	0.96 (0.55, 1.70) 0.8991	1.06 (0.58, 1.94) 0.8403
Onset to admission time, min	1.00 (1.00, 1.00) 0.4277	1.00 (1.00, 1.00) 0.0035	1.00 (1.00, 1.00) 0.1083
**Laboratory Parameters**			
RBC, ×109/L	1.00 (1.00, 1.00) 0.8319	1.00 (1.00, 1.01) 0.8823	1.00 (1.00, 1.01) 0.5542
WBC, ×109/L	1.00 (1.00, 1.00) 0.5965	1.00 (1.00, 1.00) 0.8614	1.00 (1.00, 1.00) 0.6935
Neutrophil, ×109/L	1.06 (1.01, 1.11) 0.0106	1.06 (1.01, 1.12) 0.0294	1.05 (1.00, 1.11) 0.0500
Albumin, g/L	0.98 (0.95, 1.01) 0.1621	0.97 (0.94, 1.01) 0.1098	0.97 (0.93, 1.01) 0.1694
NAR	12.11 (1.89, 77.67) 0.0086	12.44 (1.52, 101.66) 0.0187	9.34 (1.09, 80.13) 0.0417*
Lymphocyte, ×109/L	0.82 (0.70, 0.97) 0.0229	0.94 (0.81, 1.11) 0.4798	0.96 (0.81, 1.13) 0.6212
Platelet, ×109/L	1.00 (0.99, 1.00) 0.0114	1.00 (1.00, 1.00) 0.6365	1.00 (1.00, 1.00) 0.7356
INR	0.99 (0.94, 1.04) 0.5937	0.99 (0.91, 1.07) 0.7539	0.97 (0.87, 1.08) 0.5861
TC, mmol/L	1.00 (0.99, 1.00) 0.5947	1.00 (1.00, 1.01) 0.6537	1.00 (0.99, 1.01) 0.8102
TG, mmol/L	1.01 (0.98, 1.04) 0.5013	1.03 (0.98, 1.09) 0.2928	1.02 (0.95, 1.10) 0.5276
LDL, mmol/L	0.97 (0.84, 1.10) 0.6142	1.18 (1.00, 1.39) 0.0468	1.14 (0.97, 1.34) 0.1192
HDL, mmol/L	0.97 (0.92, 1.03) 0.3906	0.98 (0.89, 1.09) 0.7466	0.98 (0.86, 1.11) 0.7067
Serum glucose, mmol/L	1.03 (0.99, 1.08) 0.0964	1.02 (0.98, 1.06) 0.4038	1.02 (0.98, 1.06) 0.3583
**Treatment after Admission**			
Reperfusion therapy	-	-	-
IVT	0.69 (0.51, 0.93) 0.0164	0.54 (0.37, 0.78) 0.0010	0.46 (0.31, 0.70) 0.0002*
EVT	1.45 (1.06, 1.99) 0.0193	0.53 (0.35, 0.80) 0.0026	0.52 (0.33, 0.82) 0.0046*
Antiplatelets	0.34 (0.24, 0.48) <0.0001	0.55 (0.37, 0.83) 0.0038	0.51 (0.31, 0.83) 0.0069*
Anticoagulants	0.62 (0.43, 0.88) 0.0073	0.44 (0.29, 0.69) 0.0003	0.43 (0.26, 0.72) 0.0011*
Statins	0.33 (0.21, 0.52) <0.0001	0.41 (0.24, 0.72) 0.0019	0.67 (0.35, 1.28) 0.2216
Antihypertensive	1.17 (0.88, 1.56) 0.2683	1.16 (0.81, 1.66) 0.4185	1.30 (0.88, 1.93) 0.1909
Hypoglycemic	1.34 (0.91, 1.99) 0.1395	0.95 (0.50, 1.83) 0.8848	1.11 (0.53, 2.32) 0.7776
**HT**	6.21 (3.57, 10.81) <0.0001	3.87 (2.11, 7.12) <0.0001	3.62 (1.86, 7.05) 0.0002*

**Note:** *p value < 0.05; **#** Model 1 adjusted for age, sex, hypertension, diabetes mellitus, coronary heart disease, smoking, and baseline NIHSS score; † Model 2 adjusted for model 1 added to stroke subtypes, hemorrhage transformation, prior statin treatment, anticoagulants after admission, statins after admission, antiplatelet therapy after admission, occlusive site, and reperfusion therapy. **Abbreviations:** SBP=systolic blood pressure; DBP=Diastolic blood pressure; BMI=Body Mass Index; NIHSS= National Institute of Health Stroke Scale; TOAST=Trial of ORG 10172 in Acute Stroke Treatment; LAO=Large vessel atherosclerosis; CE=Cardioembolic; SAO=Small-artery occlusion; OE=other etiology; UE=Undetermined etiology; NAR=neutrophil-to-albumin ratio; TC=Total cholesterol; TG=triglyceride; LDL=Low-density lipoprotein; HDL=High-density lipoprotein; IVT=intravenous thrombolysis; EVT=endovascular treatment; HT=Hemorrhagic transformation; OR=odds ratio; CI=confidence interval.

## Data Availability

The datasets used and/or analyzed during the current study are available from the corresponding author upon reasonable request.

## LIST OF ABBREVIATIONS

DM: Diabetes Mellitus
EVT: Endovascular Treatment
HT: Hemorrhagic Transformation
LMR: Lymphocyte to Monocyte Ratio
NAR: Neutrophil-to-albumin Ratio
NLR: Neutrophil to Lymphocyte Ratio
ROC: Receiver Operating Characteristic

## STANDARDS OF REPORTING

STROBE guidelines were followed.
